# A single nucleotide polymorphism in the coding region of PGC-1α is a male-specific modifier of Huntington disease age-at-onset in a large European cohort

**DOI:** 10.1186/1471-2377-14-1

**Published:** 2014-01-02

**Authors:** Patrick Weydt, Selma M Soyal, G Bernhard Landwehrmeyer, Wolfgang Patsch

**Affiliations:** 1 Neurology, Ulm University, 89081 Ulm, Germany; 2 Laboratory Medicine, Paracelsus Medical University, 5020 Salzburg, Austria; 3 Pharmacology, Paracelsus Medical University, 5020 Salzburg, Austria

**Keywords:** Genetic modifier, Gender effect, Neurodegeneration, Huntington disease

## Abstract

**Background:**

Genetic modifiers are important clues for the identification of therapeutic targets in neurodegenerative diseases. Huntington disease (HD) is one of the most common autosomal dominant inherited neurodegenerative diseases. The clinical symptoms include motor abnormalities, cognitive decline and behavioral disturbances. Symptom onset is typically between 40 and 50 years of age, but can vary by several decades in extreme cases and this is in part determined by modifying genetic factors. The metabolic master regulator PGC-1α, coded by the *PPARGC1A* gene, coordinates cellular respiration and was shown to play a role in neurodegenerative diseases, including HD.

**Methods:**

Using a candidate gene approach we analyzed a large European cohort (n = 1706) from the REGISTRY study for associations between *PPARGC1A* genotype and age at onset (AO) in HD.

**Results:**

We report that a coding variant (rs3736265) in *PPARGC1A* is associated with an earlier motor AO in men but not women carrying the HD mutation.

**Conclusions:**

These results further strengthen the evidence for a role of PGC-1α in HD and unexpectedly suggest a gender effect.

## Background

Huntington disease (HD) is an autosomal-dominantly inherited, neurodegenerative disorder caused by the abnormal expansion of a CAG repeat tract in the huntingtin gene. The clinical picture of HD is characterized by a combination of a progressive movement disorder (chorea), cognitive decline and behavioral disturbances typically presenting in mid-life. However, disease onset and symptoms vary widely between individual patients. The length of the CAG repeat in the huntingtin gene and the resulting polyglutamine tract in the huntingtin protein is the major determinant of age at motor symptom onset, accounting for about 70% of the variability [1]. The remaining variability is shaped by genetic and environmental factors that remain incompletely understood [1]. Since genetic and environmental modifiers may provide clues for the identification of therapeutic targets in HD and possibly other neurodegenerative diseases, substantial research efforts are being invested into their discovery and characterization [2]. Genetic modifiers can be identified with two alternative general methodologies: the hypothesis-driven *candidate gene approach* and unbiased *genomewide screens*.

PGC-1α was identified as a candidate gene after it was discovered that PGC-1α −/− mice develop neurodegeneration and that PGC-1α dependent genes are down-regulated in human HD brains [3-7].

The transcriptional co-activator PGC-1α is a metabolic master regulator that orchestrates the adaptation of the cell to changing energy demands [8]. Originally described as regulator of mitochondrial respiration in brown adipose tissue [9], PGC-1α is now known to participate in many cell types in a wide range of essential and ancilliary metabolic processes, such as angiogenesis, antioxidative defense and autophagy. The human *PPARGC1A* gene was cloned in 1999 and subsequently found to be a genetic modifier in several metabolic diseases [10,11]. In addition to HD, PGC-1α was also implicated as an important modifier of other neurodegenerative diseases, including Parkinson disease and amyotrophic lateral sclerosis (ALS) [12,13]. The role of PGC-1α in the pathophysiology of neurodegenerative diseases is likely to be complex and differs between disease entities [14]. A route towards resolving this complexity is the detailed characterization of the *PPARGC1A* locus, its variant transcripts and SNPs that are associated with disease onset. This strategy has resulted in the identification of a novel promoter, new brain specific PGC-1α isoforms and new variants associated with HD age at symptom onset [15].

In HD, independent analysis of three European HD cohorts suggested a protective effect of SNP 7665116 on age of onset in HD [16-18]. Further SNPs, for which an effect on AO was reported, include rs2970870, rs2970848 and rs6821591 [15,19,20]. These SNPs all map to non-coding portions of the gene.

As non-synonymous SNPs may be associated with functional changes of the protein, we have previously studied associations of rs8192678, the most frequent SNP in the coding region (minor allele frequency ~0.35) with HD AO, but found no associations in a smaller Italian population [16]. As the current population is much larger and therefore increases the power to detect effects of less common SNPs, we studied the next common coding SNP, rs 3736265 (minor allele frequency ~0.05). Here we report, that this SNP is significantly associated with AO and that this association is male-specific.

## Methods

We analyzed DNA samples and clinical data provided by the European HD network REGISTRY study, one of the largest cohorts available for this kind of study [21]; (http://www.euro-hd.net). In DNA from 1706 unrelated symptomatic HD patients (869 men/837 women), rs3736265 was typed using a TaqMan Genotyping Assay (C_25935762_20, Applied Biosystems). Correct genotyping was verified in 10% of the samples. Allele frequencies were estimated by gene counting. Agreement with Hardy-Weinberg equilibrium was ascertained using a χ^2^ goodness-of-fit test. Association of rs3736265 with motor AO was ascertained in a linear model. As we observed a skewed relationship between the expanded CAG repeat size and age of onset as expected, we used logarithmically transformed AO as the dependent variable and rs3736265 (coded as 0, 1 and 2) and normal and expanded CAG repeat sizes as well as their interactions as independent variables (Duyao M et al. 1993, Soyal SM et al. 2012). We observed a linear relationship between HD CAG repeat size and log-transformed AO. Furthermore, the residuals appeared to follow a normal distribution as judged by the Kolmogorov-Smirnov test. The access to the databases used in our work is publicly available. Participants gave informed written consent according to the International Conference on Harmonisation-Good Clinical Practice (ICH-GCP) guidelines (http://www.ich.org/LOB/media/MEDIA482.pdf). For participants, who lacked capacity to consent, study sites adhered to country-specific guidelines for obtaining consent. Minors assented with both parents consenting for them. Ethical approval was obtained from the local ethics committee for each study site contributing to REGISTRY [21].

## Results and discussion

The results of the genotyping are summarized in Table 1. The distribution of genotypes associated with rs3736265 met Hardy-Weinberg (HW) expectations. The minor allele frequency (MAF) is 0.0554. 186 of the 1706 samples (10.1%) were heterozygous and two samples were homozygous for the minor allele. In the overall population analysis there was no significant effect in the additive model, but a significant effect in the dominant model (p = 0.0519 and 0.0170, respectively). Gender stratification revealed that the SNP effect was observed in the male population and both the additive and the dominant model reached significance in men after the Bonferroni correction (p = 0.0328 and 0.0084, respectively). The difference in AO between male patients harboring two major alleles and a minor allele was ~2.6 years, while in women AO was not significantly affected.

**Table 1:** Effect of PPARGC1A rs3736265 on AO

	**rs3736265 genotype**			**P**^**1**^	**P**^**2**^
	**CC**	**CT**	**TT**		
All					
N	1518	186	2		
HD CAG repeat size	44.7 (4.6)	44.7 (3.8)	46.0 (2.8)	n.s.	n.s.
non-HD CAG repeat size	18.5 (3.4)	18.5 (3.3)	19.0 (2.8)	n.s.	n.s
CAG-product^3^	828 (178)	831 (185)	870 (76)	n.s.	n.s.
Log AO^4^	1.641 (0.128)	1.627 (0.126)	1.601 (0.088)	0.0519	0.0170
AO^5^	43.75	42.36	39.9		
Men					
N	784	84	1		
HD CAG repeat size	44.8 (4.5)	44.5 (.2)	44.0 (0)	n.s.	n.s.
non-HD CAG repeat size	18.6 (3.5)	18.8 (3.5)	21.0 (0)	n.s.	n.s
CAG-product^3^	836 (185)	840 (176)	924 (0)	n.s.	n.s.
Log AO^4^	1.655 (0.128)	1.629 (0.126)	1.638 (0)	0.0164	0.0042
AO^5^	45.19	42.56	43.45		
Women					
N	734	102	1		
HD CAG repeat size	44.5 (4.7)	44.8 (4.2)	48.0 (0)	n.s.	n.s.
non-HD CAG repeat size	18.4 (3.4)	18.3 (3.2)	17.0 (0)	n.s.	n.s.
CAG-product^3^	818 (170)	823 (192)	816	n.s.	n.s.
Log AO^4^	1.626 (0.128)	1.622 (0.127)	1.568 (0)	0.6732	0.5705
AO^5^	42.27	41.88	36.98		

Data are means (SD); P^1^, additive model; P^2^, dominant model; ANOVA; ^3^product of the CAG sizes in the two alleles ^4^adjusted for HD CAG and non-HD CAG sizes and their products. ^5^ANOVA was used and the p-values refer to the log-transformed age-at-onset. The age-at-onset is only shown to give an estimate of the effects that is easier to interpret. All genotype distributions are in HW equilibrium, MAF in entire population 0.0554.

The fact that rs3736265 is a low frequency SNP implies that small differences in analysis might affect the significance of the association. We therefore scrutinized the robustness of the association. First, three men were homozygous, possessing HD alleles with 54 and 37, 50 and 37 or 44 and 36 CAG repeats. Exclusion of these subjects from the analysis showed p-values of 0.0040 and 0.0036 for rs3736265 in the additive and dominant model, respectively. Second, differences in the relationship between CAG repeat length and AO were noted between the sixteen study centers. Adjustment for these differences did not affect the significance of the association of rs3736265 with logarithmically transformed AO (p-values 0.0047 and 0.0044 for additive and dominant model, respectively). Third, according to a previous study, the normal CAG repeat has no effect on AO [22]. We therefore omitted the normal allele and its product with the HD allele from the analysis and found similar p-levels (0.0037 and 0.0034). Fourth, few samples at the end of the distribution with large repeats may disproportionally drive the results obtained. To address this possibility, we omitted the 21 subjects with HD-CAG size > 55 and observed similar p-values as in the entire male cohort (0.0044 and 0.0040). Additional exclusion of 12 patients with HD-CAG size <40 resulted in p-values of 0.0075 and 0.0070. We also addressed the issue of modifying effects in women with <55 CAG repeats and found no modifying effect of rs3736265 (P-values 0.882 and 0.843 for the additive and dominant models). Finally, the collection of subjects from different European regions and in different centers is likely to result in a reduced signal-to-noise ratio compared to smaller single-region or single-center studies. Hence, the association observed is robust and fulfills the expectations of linear models as linear relationships between variable and a normal distribution of residuals were observed.

The *PPARGC1A* genomic locus is of potential importance for understanding the mechanisms of PGC-1α action in HD pathogenesis. We thus examined this locus in more detail and demonstrate that a SNP in the coding region of *PPARGC1A* has a significant effect on age of onset in HD.

SNP rs3736265 is located in exon 9 of *PPARGC1A*, hence contained in many different tissue specific PGC-1α isoforms reported to date, i.e. PGC-1α1-4 and PGC-1αB1-5 [15,23]. It results in a Thr612Met amino acid exchange. Hence, a polar, aliphatic amino acid (threonine) is substituted by a sulphur-containing hydrophobic, aliphatic amino acid (methionine). The amino acid stretch comprising the more common threonine is predicted to have a random coil or extended strand structure. The substitution by methionine is likely to result in an α-helical structure of five amino acids [24]. Co-activation of PPARγ on the acyl-CoA-binding promoter did not differ between PGC-1α proteins harboring threonine or methionine at amino acid residue 612 [25]. However, a recent study reported significant associations of rs3736265 with neovascularization in age-related macular degeneration (AMD) as well as interactions with the AMD-associated genes encoding complement factor B and VEGFA [26].

Surprisingly, the described SNP effect is clearly male-specific, while HD - unlike other neurodegenerative diseases - does not show a gender bias. This is reflected in our study population with a balanced male to female ratio (1.04). Some gender differences in HD are emerging: In the REGISTRY population women tend to have poorer motor and functional scores and a faster rate of disease progression than matched male controls [27]. This however did not translate into differences in age at symptom onset. Mochel et al. found a lower body mass index (BMI) and lean body-mass despite a higher caloric intake in men but not in women [28]. Similarly, a transgenic rat model of HD displayed a marked male-specific gender-bias of symptoms [29]. This study includes a gene-expression profiling study on human HD striata showing only 42 genes to be differentially regulated between men vs. women with HD, compared to 343 genes differentially regulated between male vs. female controls. A possible explanation for the weakness of this signal is that the 1.5 fold-change cut-off in the analysis is too strict to allow for PGC-1α connected changes to become evident [5]. Thus, a net-work based analysis may have been superior to a bioinformatics approach that looks at each transcript in isolation. Finally, sex-specific influences of genetic modifiers in HD have been reported for apolipoprotein E [30] and for the NR2A and NR2B receptor genes [31], but these results were not confirmed in other studies [32,33]. An important difference between these studies and our present work is that our HD cohort is larger by an order of magnitude (n = 138 and n = 250 vs. n = 1706, respectively). Nevertheless, the association we report here awaits independent confirmation.

## Conclusions

In summary our result further strengthens the accumulating evidence for a role of the PGC-1α-pathway in HD in particular and neurodegeneration in general and underlines that gender differences in neurodegenerative diseases might be due to differences at the molecular level [34].

## Competing interests

All authors report no conflicts of interest. Appropriate approval and procedures were used concerning human subjects.

## Authors’ contributions

PW participated in the design of the study and drafted the manuscript. SMS designed and carried out the molecular genetic investigation strategy and contributed to the manuscript. GBL participated in the design of the study and the drafting of the manuscript. WP participated in the design of the study, performed the statistical analysis and drafted the manuscript. All authors read and approved the final manuscript.

## Pre-publication history

The pre-publication history for this paper can be accessed here:

http://www.biomedcentral.com/1471-2377/14/1/prepub
